# Enhanced adsorptional-photocatalytic degradation of chloramphenicol by reduced graphene oxide-zinc oxide nanocomposite

**DOI:** 10.1038/s41598-022-21266-5

**Published:** 2022-10-12

**Authors:** K. O. Sodeinde, S. O. Olusanya, O. S. Lawal, M. Sriariyanun, A. A. Adediran

**Affiliations:** 1 Materials and Nanoresearch Unit, Department of Industrial Chemistry, Federal University, Oye-Ekiti, Ekiti State Nigeria; 2 Biorefinery and Process Automation Engineering Center, The Sirindhorn International Thai-German Graduate School of Engineering, King Mongkut’s University of Technology North Bangkok (KMUTNB), Bangkok, Thailand; 3 Materials Design and Structural Integrity Group, Department of Mechanical Engineering, Landmark University, Omu-Aran, Kwara State Nigeria

**Keywords:** Environmental sciences, Materials science, Nanoscience and technology

## Abstract

Improper discharge of waste dry cell batteries and untreated antibiotics laden effluents to the environment pose serious threat to the sustenance of the ecosystem. In this study, synthesis of reduced graphene oxide-ZnO (rGO-ZnO) nanocomposite was achieved via a bioreduction process using waste dry cell battery rod as graphene oxide (GO) precursor. The nanocomposite was applied in the ultraviolet photocatalytic degradation of chloramphenicol (CAP) at 290 nm in the presence of hydrogen peroxide. RGO-ZnO nanocomposite was characterized by SEM, TEM, XRD, BET and FTIR. TEM image of the nanocomposite revealed a polydispersed, quasi-spherical zinc oxide on a coarse reduced graphene oxide surface. XRD patterns showed sharp, prominent crystalline wurtzite hexagonal phases of ZnO and rGO. BET surface area of the nanocomposite was 722 m^2^/g with pore size of 2 nm and pore volume of 0.4 cc/g. % photo-removal efficiency increased with increasing irradiation time but diminished at higher pH, temperature and CAP concentration. Photocatalytic adsorption process fitted more accurately into the Freundlich model (R^2^ = 0.99) indicating a multilayer adsorption mechanism. 92.74% reduction in chemical oxygen demand (COD) level of veterinary effluent was obtained after treatment with the nanocomposite thus affirming its effectiveness in real waste water samples.

## Introduction

Since their initial discoveries several decades ago, antibiotics have found useful applications in human and animal care for disease prevention, treatment and growth enhancement purposes^1^. Various antibiotics that have been in use for these purposes include azithromycin, chloramphenicol, tetracycline, streptomycin, etc. However, in recent years, antibiotics and their residues have entered the environment via production plants, waste water treatment plants, hospitals, landfills, etc. where they have now emerged as important group of environmental pollutants^2^. Different levels of antibiotic residues have been reported in pharmaceutical and agricultural effluents with their attendant toxic effects on aquatic and terrestrial organisms^2^.

Advanced oxidation processes such as semiconductor-based homogenous/heterogenous photocatalysis, sonocatalysis, fenton, electrochemical oxidation, ozonation, etc. have gained enormous attention over the last few decades for the treatment of pharmaceutical and agricultural effluents due to their numerous advantages including cost effectiveness, time and ability to degrade organic and inorganic pollutants to less toxic substances^3–7^. Semiconductors such as cadmium sulfide (CdS), zinc sulfide (ZnS), ferric oxide (Fe_2_O_3_), titanium oxide (TiO_2_) and zinc oxide (ZnO) nanoparticles can function as a sensitizer for light reduced redox reactions via the generation of reactive oxidative species including hydroxyl radicals, hydrogen peroxide and superoxide anions^8^.

Zinc oxide is an II-VI compound semiconductor. It possesses a band-gap of 3.4 eV and a wavelength of 387 nm and can be excited in the ultraviolet (UV) region of the electromagnetic spectrum^9^. Zinc oxide nanoparticles has been widely used in antimicrobial agent, gas sensing applications and most importantly in photocatalytic waste water treatment due to its relatively cheap cost of production, mild reaction conditions^10,11^, etc.

Conversely, some problems often encountered with semiconductor photocatalysts include narrow absorption range, photocorrosion, recombination of electron–hole pair, photo-instability in aqueous medium, wide band gap necessitating huge activation energy, etc^11,12^. Integration of graphitic materials (reduced graphene oxide) has been proved to be effective for preventing the recombination of electron–hole pairs, enhancement of transportation of charge carriers in semiconductors, improved adsorption capacity, conductivity and overall photocatalytic activities^13,14^.

Various graphene oxide based photocatalysts have been reported for the treatment of effluents^15^. For instance, Rakkappan and Halliah^16^ carried out one-step hydrothermal synthesis of Pd nanoparticles with rGO-ZnO nanocomposites and investigated its antimicrobial, antioxidant, cytotoxic activities. The Pd-rGO-ZnO nanocomposite was prepared without using any chemical reductants. The results indicated good biocompatibility and excellent performance towards biological activities. Met et al.^17^ prepared reduced graphene oxide–zinc oxide (rGO-ZnO) by rapid microwave-assisted hydrothermal technique. The nanocomposite was applied in the photodegradation of methyl orange (MO) dye from water under UV light. Investigation revealed that the incorporation of rGO into the ZnO increased the photodegradation ability of the bare ZnO. RGO obtained from Tang Lau method formed more stable and efficient composite with ZnO and exhibited higher activity compared to the rGO composite prepared from conventional Hummer's method. Naseem et al.^18^ synthesized reduced graphene oxide/zinc oxide (rGO/ZnO) nanocomposite using a chemical method. The rGO/ZnO nanocomposite was analyzed for the reduction of methylene blue (MB), 4-nitrophenole (4-NP) and its antibacterial activity. The degradation rate of MB under UV lamp by rGO/ZnO nanocomposite was found to be 80% after 5 h. The reduction of 4-NP into 4-aminophenol (4-AP) was much faster in the presence of reducing agent (NaBH_4_) than without it. The results of antibacterial activity showed that rGO/ZnO nanocomposite exhibits better antibacterial activity than GO.

Chloramphenicol (CAP); an effective broad‐spectrum antibiotic with excellent antibacterial properties, has been widely used to inhibit both gram‐positive and gram‐negative bacteria in humans and other mammals^19^. Excessive usage of CAP may result in bone marrow depression, fatal aplastic anaemia, gray baby syndrome in humans, etc^20^. Consequently, CAP has been banned by many developed countries for use in food‐producing animals. However, CAP is still widely used in many developing countries due to its low cost and availability, leading to water and soil pollution and increase in microbial resistance^2^.

Due to the fast growing demand for various electronic devices such as power tools, digital cameras, lamps, remote controls, electronic readers, etc., the volume of spent dry cell batteries has been increasing at a high rate in recent years. Waste dry cell batteries are environmental nuisance and hazardous in nature particularly in developing countries where recycling is a major challenge. Indiscriminate discharge into soil and waterways pose serious threat due to their poor biodegradability. Waste dry cell battery graphite rods can be utilized as an economically viable precursor for graphene oxide (GO) in the ultimate synthesis of reduced graphene oxide-zinc oxide nanocomposite photocatalyst. Hence, the work focused on the green synthesis, characterization of reduced graphene oxide-zinc oxide nanocomposite using waste battery graphite rods and aqueous extract of *Amaranthus cruentus* as GO precursor and reducing agent respectively. In addition, the application of the nanocomposite photocatalyst in the ultraviolet (UV) photodegradation of CAP at 290 nm under different reaction conditions and practical application in veterinary effluent treatment are herein reported.

## Experimental

### Materials/Reagents

**Figure 1:**
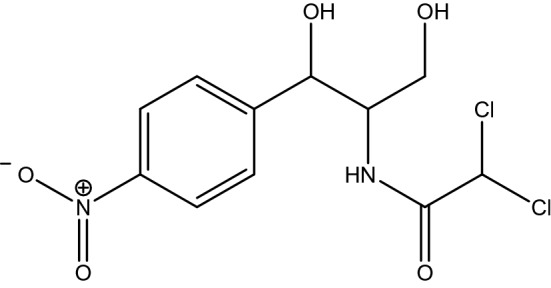
Structure of chloramphenicol (M.W. = 323.13 g/mol).

Chloramphenicol (Fig. 1) and zinc nitrate were supplied by Loba Chemie (India). Concentrated sulphuric acid (99.9%), potassium tetraoxomanganate (VII), hydrogen peroxide and ethanol were purchased from Merck (UK). All the chemicals used were of analytical reagent grade or the highest purity available and were used as received.

### Sample collection and treatment

*A. cruentus* leaves were purchased from a local market in Oye-Ekiti, Ekiti State. Twenty (20) 1.5 V waste dry cell batteries (Tiger brand) were randomly collected from domestic/office electronic wastes in Oye-Ekiti, Ekiti State, Nigeria. The graphite electrodes were carefully removed from the waste batteries and ground with a mechanical grinder. The pulverized graphite rod was kept in a glass vial and stored at room temperature (30 ± 2 °C) till further use. 100 g/L stock solution of CAP was prepared in distilled water and serial standard solutions (1–10 g/L) were prepared by corresponding dilution of the stock solution with distilled water.

### Preparation of graphene oxide from powdered graphite rods

Graphene oxide (GO) was prepared from powdered graphite rod by modified Hummer’s method^21^. Briefly, 1 g of powdered graphite rod was carefully weighed into a 250 mL flat bottom flask placed in an ice-water bath. 23 mL concentrated sulphuric acid was added stirred for 10 min. 3 g of KMnO_4_ was added and the solution stirred for 10 min and left inside the water bath for 1 h. The temperature was increased to 30 °C and stirred for 1 h. 46 mL distilled water was then added dropwise and the temperature raised to 96 °C for 30 min. 10 mL H_2_O_2_ was added to the solution followed by 140 mL distilled water. GO was obtained by centrifugation at 3500 rpm for 10 min and washed with methanol followed by acetone. The GO residue was oven dried at 80 °C for 4 h and kept in a sealed glass vial until further use.

### Preparation of aqueous extract of *A. cruentus*

*A. cruentus* leaves were air-dried in the room and washed thoroughly with distilled water. 10 g of finely cut leaves were boiled in 150 mL distilled water for 20 min and extract filtered with a filter paper. The filtrate was cooled to room temperature and refrigerated at 4 °C.

### Preparation of rGO-ZnO nanocomposite using aqueous extract of *A. cruentus*

**Figure 2:**
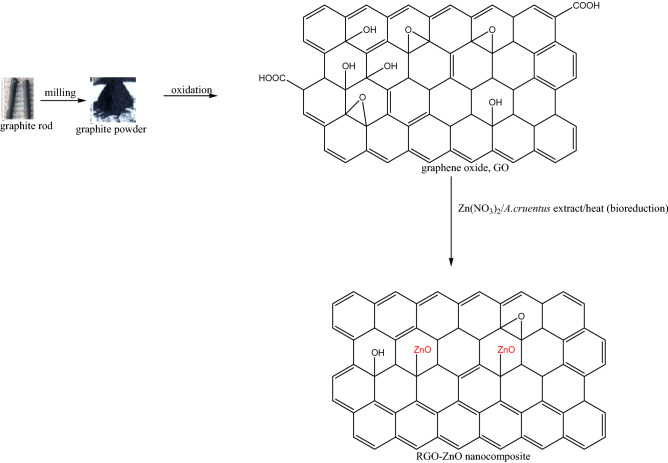
Synthesis mechanism of rGO-ZnO nanocomposite.

The synthesis of rGO-ZnO nanocomposite was achieved following the method of Sandya et al.^22^ with slight modification. 1 g of GO was treated with 100 mL 5 mM Zn(NO_3_)_2_ solution and 100 mL of freshly prepared aqueous extract of *A. cruentus* (as the reducing agent) in a 500 mL flat bottom flask. The mixture was heated at 80 °C on a magnetic stirrer with constant stirring at 300 rpm for 45 min to ensure simultaneous bioreduction of Zn(NO_3_)_2_ and GO. The resulting rGO-ZnO nanocomposite was cooled to room temperature and centrifuged at 3500 rpm for 10 min (Fig. 2). The nanocomposite was washed with methanol, followed by acetone. It was finally oven dried at 80 °C for 4 h and kept in a sealed glass vial until further use.

### Scanning electron microscopy (SEM) analysis

Surface morphological evaluation of rGO-ZnO nanocomposite was carried out using scanning electron microscopic tool. A thin layer of the sample granule was placed on aluminium specimen holder by double-sided tape. The sample was sputter-coated with gold to a thickness of about 30 nm to prevent charging. The specimen holder was transferred to XL-20 series. Scanning electron microscope of rGO-ZnO nanocomposite was examined using a JEOL USA Model: JSM-7900 F at an accelerating voltage of 15–20 kV.

### FTIR spectroscopic analysis

FTIR spectra of dried *A. cruentus* leaves (2 mg) were run as KBr pellets on Shimadzu Spectrum TwoTM spectrometer in the frequency range 4000–400 cm^−1^.

### Wide angle X-ray diffractometry

The crystalline phases of the raw powdered graphite and rGO-ZnO nanocomposite samples were obtained using Empyrean XRD diffractometer at 40 mA and 45 kV equipped with Cu Kα (1.5418 Å) radiation at an angular incidence 2θ range of 10–75°.

### Brunauer–emmett–teller (BET) surface area analysis

The specific surface area (BET) of rGO-ZnO nanocomposite was measured with a Quantachrome instrument using the adsorption of N_2_ at the temperature of liquid nitrogen. Prior to analysis, the sample was degassed at 523 K for 3 h. The pore size and pore volume were estimated using Barrett-Joyner-Halenda (BHJ) theory.

### Photocatalytic degradation studies

The photocatalytic activity of rGO-ZnO nanocomposite in the photodegradation of CAP was investigated in batch mode. The effect of variation in temperature was studied at 30, 40, 50, 60 and 70 °C. 0.1 g of rGO-ZnO nanocomposite was added to a mixture containing 2 mL 30% hydrogen peroxide and 40 mL 1.0 g/L CAP solution in a 200 mL Pyrex glass beaker. The mixture was stirred continuously on a magnetic stirrer for 30 min at 300 rpm in the dark to ensure equilibration at 30 °C. The solution mixture was carefully transferred into a flat dish with a 100 mm diameter and exposed to ultraviolet (UV) radiation using a UVP Compact lamp (4 W, 230 V, λ = 254 nm) for 40 min. The distance between the UV source and the photo-reaction vessel was 10 cm. The procedure was repeated at 40, 50, 60 and 70 °C. The influence of pH on the photocatalytic degradation process was studied between pH 2–10 by adjusting with 0.1 M HCl or NaOH. The effect of irradiation time was monitored at 20, 40, 60, 80 and 100 min. The CAP concentration was investigated at 1–10 g/L. For the effect of dosage, a suspension was prepared by adding 0.1–1.0 g of rGO-ZnO nanocomposite to a mixture containing 2 mL 30% hydrogen peroxide and 40 mL 1 g/L CAP solution in a 100 mL Pyrex glass beaker. The mixture was stirred continuously on a magnetic stirrer for 30 min at 300 rpm in the dark to ensure equilibration at room temperature. In each case, the resulting mixture was filtered to separate the photocatalyst and absorbance of the filtrate measured at 290 nm using a T-60 UV–Visible spectrophotometer. The photo-removal efficiency experiments were carried out for four repeated cycles to study the recyclability and stability of the rGO-ZnO nanocomposite sorbent. After each cycle, the nanocomposite catalyst was filtered, washed with distilled water and dried in the oven at 105 °C before reuse. The photo-removal efficiency percentage was calculated using^23^

1 $$ \% {\text{ Photo}} - {\text{removal efficiency of CAP }} = \frac{{C_{{\text{o}}}  - C_{f} }}{{C_{{\text{o}}} }}~ \times ~100~ $$

where C_0_ is the initial concentration of CAP and C_f_ is the concentration of CAP after photo-irradiation. The amount of CAP adsorbed; q_e_ (*mg* of CAP per *g* rGO-ZnO) was calculated according to Ekhrami et al.^23^

2 $$ q_{e}  = ~\frac{{{\text{C}}_{{\text{o}}}  - {\text{C}}_{{\text{e}}} }}{{\text{W}}}~~{{ \times }}~V $$

where C_e_ is the equilibrium concentration of analyte, V (mL) is the volume of the solution, W(g) is the mass of CAP, q_e_ (mg/g) is the amount adsorbed. For practical application of the effectiveness of the nancomposite photocatalyst, effluent containing CAP from a local farm house was collected in a glass bottle and analysed as previously stated before and after treatment with 0.1 g of rGO-ZnO nanocomposite. The chemical oxygen demand (COD); a measure of the amount of dissolved oxygen required for complete oxidation of organic pollutants, was equally determined before and after treatment using standard method^24^.

### Adsorption isotherm and kinetic models

Both Langmuir and Freundlich adsorption isotherm models were adopted. The Langmuir isotherm is based on the theoretical assumption that the adsorption consists entirely of a monolayer at the surface. The heat of adsorption does not depend on the number of sites and is equal for all sites. The linearised form of the Langmuir model as described by Sodeinde et al.^25^ is shown in Eq. (3).

3 $$ {\text{1/q}}_{{\text{e}}} {{~ = 1/(q}}_{{\text{m}}} {\text{K}}_{{\text{L}}} {{~) \times 1/C}}_{{\text{e}}} {{~ + 1/q}}_{{\text{m}}}  $$

where *Ce* is the equilibrium concentration of dye solution (mg/L), *q*_*e*_ is the amount of pollutant adsorbed per unit mass of the rGO-ZnO photocatalyst (mg/g), *q*_*m*_ is the Langmuir constant representing adsorption capacity (mg/g), and *K*_*L*_ is Langmuir constant representing energy of adsorption (L/mg). A plot of 1/q*e* against 1/C*e* is linear for a sorption process obeying the basis of this equation with *k*_*L*_ and *q*_*m*_ obtained from the slope and intercept respectively.

The Freundlich isotherm describes the heterogeneity of the adsorbent surface by multilayer adsorption of analyte and non-uniform distribution of the heat of adsorption over the adsorbent surface. The linear form of the Freundlich equation as described by Igwe and Abia^26^

4 $${\text{log}} \, {\text{q}}{\text{e}} \, \text{=} \, {\text{log}} \, {\text{K}}{\text{F}} \, \text{+1/n} \, {\text{log}} \, {\text{Ce}}$$

where *C*_*e*_ is the equilibrium concentration of CAP solution (mg/L), *q*_e_ is the amount of pollutant adsorbed per unit mass of the rGO-ZnO nanocomposite photocatalyst (mg/g), *n* is the number of layers, and *K*_*F*_ is the Freundlich constant. A straight line plot of log q_*e*_ against log *C*_*e*_ has *K*_*F*_ and *n* as the intercept and slope respectively. Lagergren pseudo-first-order model^27^ and pseudo-second-order kinetic model^28^ were adopted for detailed kinetic studies as described in Eqs. (5a) and (5b) respectively.

5a $$ln({q}_{e}-{q}_{t}) = ln {q}_{e}-{k}_{1}t$$

5b $$t/qt =1/({k}_{2}.{q}_{e}^{2}) + t/{q}_{e}$$

where q_t_ and q_e_ are the amount of pollutant adsorbed (mg/g) at time t and equilibrium, respectively. k_1_ (min^-1^) is the rate constant of the pseudo-first-order kinetic model and k_2_ (g.mg^-1^.min^-1^) is the rate constant of the pseudo-second-order kinetic model.

## Results and discussion

### Result of FTIR analysis of *A. cruentus* leaves

**Figure 3:**
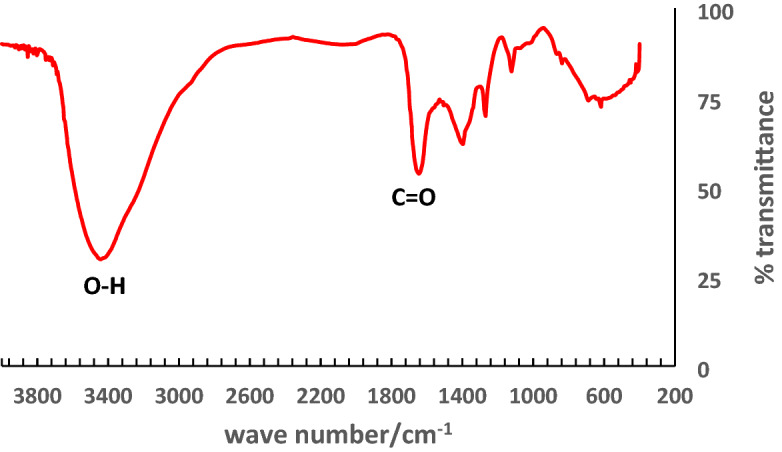
FTIR spectra of *A. cruentus* aqueous extract.

The result of the FTIR analysis of the *A. cruentus* aqueous extract is shown in Fig. 3. The peaks observed at 1637.46 and 3450.82 cm^−1^ correspond to C = O carbonyl and –OH stretching vibrations respectively. The peaks at 1211.34, 1126.47 and 1435 cm^−1^ showed the stretching vibration of C−O (alkoxy/alkoxide group). Phytochemical screening of the extract confirmed the presence of terpenoids, flavonoids, carbohydrates and saponins. The functional groups in the phytochemicals are responsible for the bioreduction of GO and zinc salt to rGO-ZnO nanocomposite. We had earlier established in our previous studies the roles of biomolecules in the biosynthesis of ZnO nanoparticles as reducing, capping and stabilizing agents^10^.

### Result of SEM and TEM analyses

**Figure 4:**
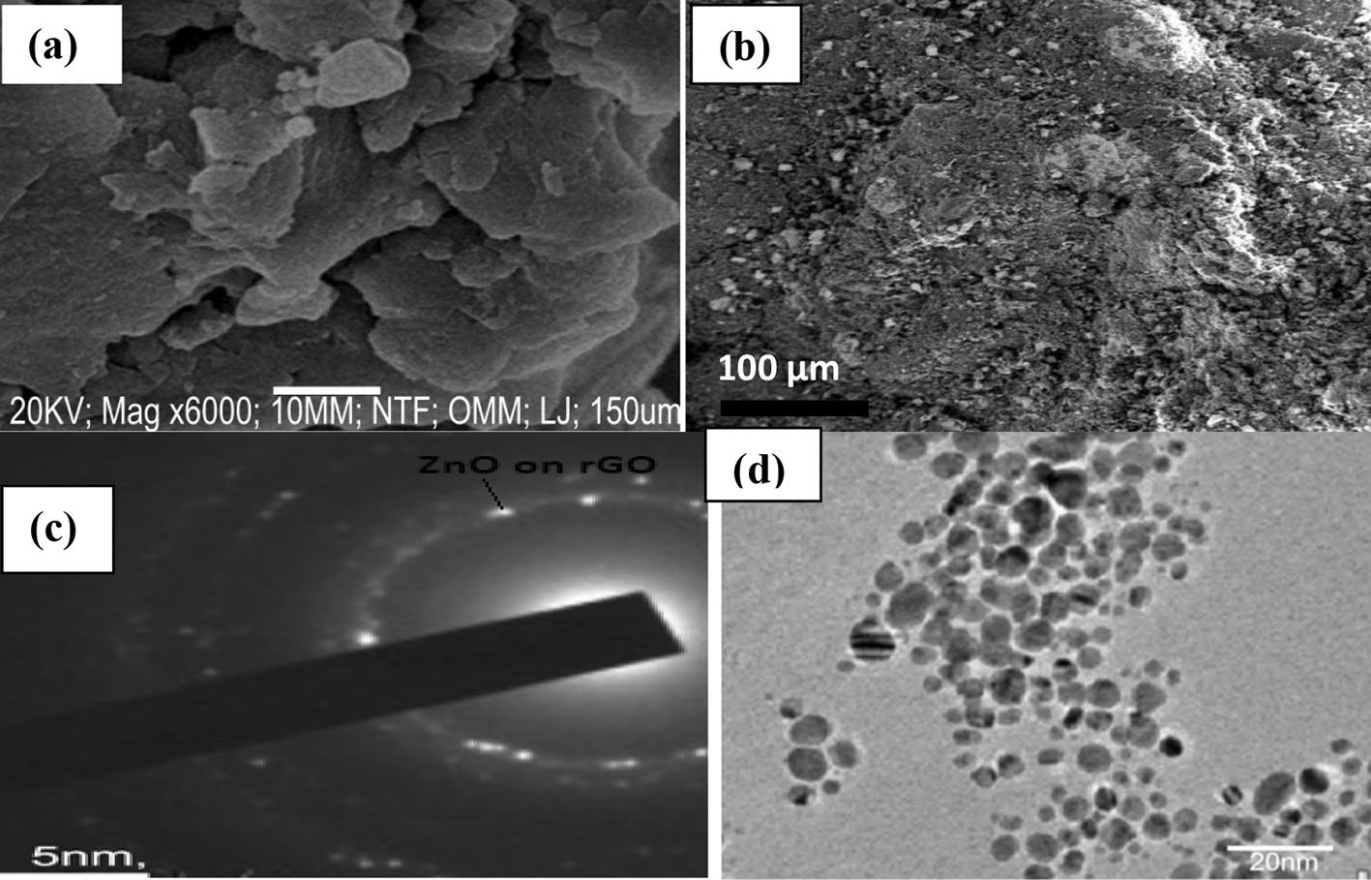
SEM image of (**a**) rGO, (**b**) rGO-ZnO nanocomposite, (**c**) TEM micrograph with selected area electron diffraction (SAED) of rGO-ZnO nanocomposite, (**d**) TEM image of rGO-ZnO nanocomposite.

The SEM micrographs of as-synthesized rGO and rGO-ZnO nanocomposite are shown in Fig. 4a,b respectively. The SEM image of rGO showed a smooth, flake-like morphology (Fig. 4a). The incorporation of ZnO into rGO revealed a polydispersed, quasi-spherical ZnO NPs on the rough reduced graphene oxide sheet surface (Fig. 4b). The selected area electron diffraction (SAED) patterns from the nanocomposite showed the intense diffraction spots indicating that the ZnO NPs were very well-crystallized (Fig. 4c). The rough nature of the reduced graphene oxide matrix facilitates facile adsorption of analytes onto its surface. In order to complement the SEM morphology, the microstructure of the nanocomposite was further subjected to TEM analysis. Figure 4d affirmed the irregular, quasi-spherical nature of ZnO NPs on the rGO surface. The particle size range of the nanocomposite is between 8 and 14 nm. The ZnO nanocrystals on the rGO surface are obtained by the bioreduction of the Zn(NO_3_)_2_ in aqueous extract of *A. cruentus* at 80 °C for 45 min. Zinc dissociates into Zn^2+^ ions and the hydroxyl ion (–OH) functional group in the extract reacted with the Zn^2+^ ions to form zinc hydroxide ^17^ which further decomposed to ZnO NPs. Upon nucleation, ZnO nanocrystal growth on rGO surface is by coalescence of adjacent particles that share a common crystallographic orientation thus suggesting an oriented attachment (OA) growth mechanism. Careful control of rGO-ZnO nanocomposite morphology was achieved by adjusting the temperature and ensuring mild stirring. The equation of the reaction is given as:

$$ Zn\left( {NO_{3} } \right)_{2} \underset {} \longleftrightarrow Zn^{2 + } + 2NO_{3}^{ - } $$

$$ Zn^{2 + } + 2OH^{ - } \left( {\text{from extract}} \right)\underset {} \longleftrightarrow Zn\left( {OH} \right)_{2} $$

$$ Zn\left( {OH} \right)_{2} \underset {} \longleftrightarrow 2ZnO + H_{2} O $$

### Result of wide angle X-ray diffraction patterns of raw graphite and rGO-ZnO nanocomposite

**Figure 5:**
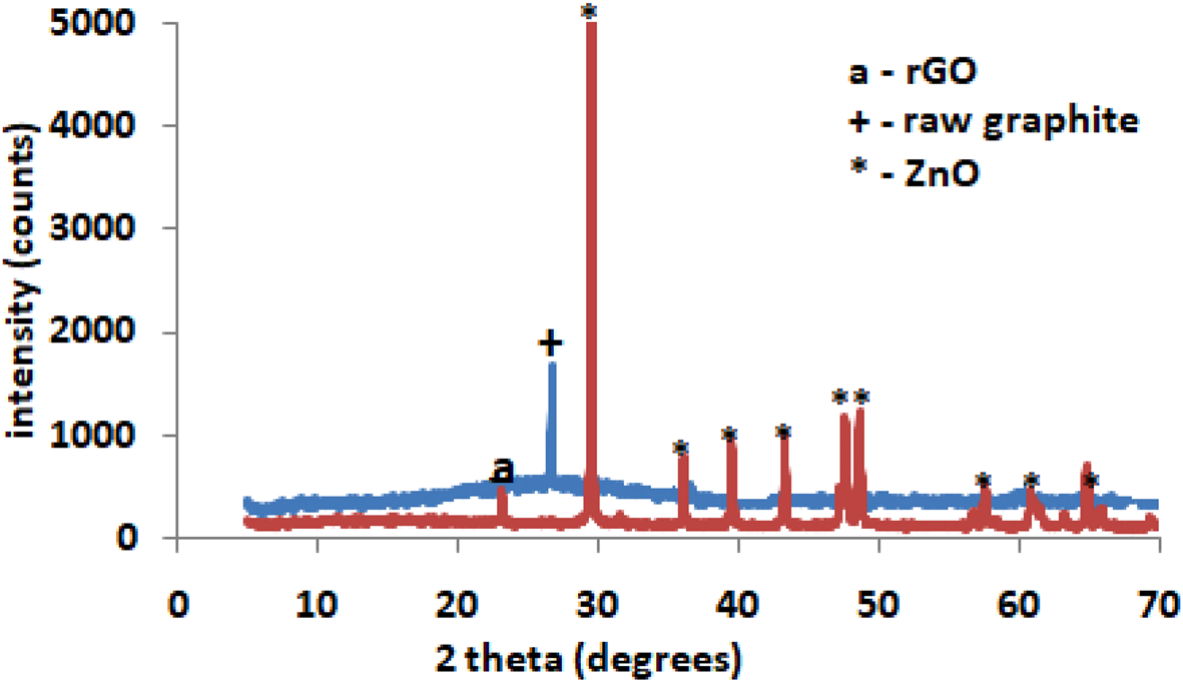
XRD patterns of raw graphite and rGO-ZnO nanocomposite.

X-ray diffraction is a powerful tool for the determination of phase composition and structural crystallinity of both raw graphite and rGO-ZnO nanocomposite. In the case of raw graphite rod powder (Fig. 5), the presence of only one diffraction peak around 2*θ* = 28° which correspond to the (001) plane of graphite affirmed its structural integrity and composition^29^. The peak around 2*θ* = 23.8° corresponds to the (002) plane of the reduced graphene oxide in the nanocomposite^29^. The main peaks at 2*θ* angles of 30.13, 35.41, 43.13, 47.83, 49.16, 58.14, 62.45, 64.52 and 70.12° correspond to the crystallographic planes of (201), (112), (200), (103), (110), (102), (101), (002), (100) respectively of Wurtzite hexagonal phases of ZnO. This diffraction pattern agreed with the standard JCPDS card No. 00-036-1451 data and other previous studies for ZnO nanoparticles^3,29,30^. The average crystallite size of the nanocomposite was calculated using the Debye–Scherrer Eq. (6) as follows:

6 $$ D = K\lambda /({{\beta Cos}}\theta ) $$

where D is the crystallite size, K is Scherrer constant (0.89), λ is X-ray wavelength (0.154 nm), $\upbeta$ is the peak full width at half maximum (FWHM) in radian, and θ is the Bragg diffraction angle. The crystallite size for rGO-ZnO nanocomposite was about 12.4 nm.

### Result of BET surface analysis of rGO-ZnO nanocomposite

**Figure 6:**
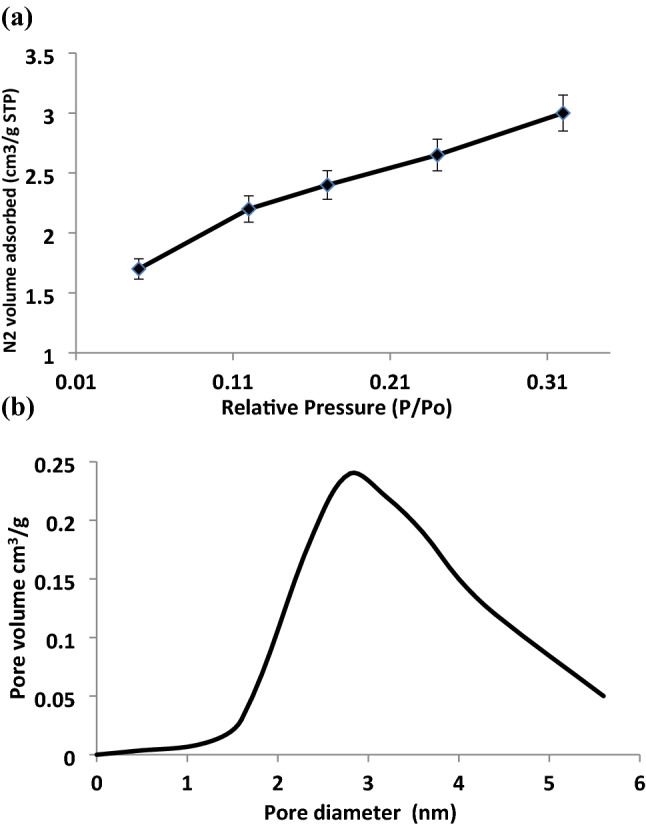
Nitrogen adsorption/desorption isotherm (**a**), BJH pore size distribution (**b**).

Mesoporosity of the nanocomposite photocatalyst is a major factor in facilitating rapid electron transfer, higher specific surface area for pollutants adsorption and mass transportation to the reactive site and overall enhancement of the photodegradation process^30^. The N_2_ adsorption–desorption isotherm of the rGO-ZnO nanocomposite is shown in Fig. 6a. The BET surface area of the synthesized rGO-ZnO nanocomposite is 722 m^2^/g. The BJH surface area was measured to be about 617 m^2^/g. The pore size distribution curve (Fig. 6b) showed the pores of rGO-ZnO nanocomposite were mostly located in the mesoporous (2–6 nm) range with an average pore size of 2 nm. It is observed that the isotherm is of type IV and the hysteresis loop is of the H_2_ type according to the IUPAC classification thus affirming its mesoporous nature. The BJH cumulative pore volume was calculated to be about 0.4 cc/g. The result of this study revealed a much improved surface area and narrower pore size than previous works involving ZnO nanoparticles alone^3,31^.

### Result of photocatalytic studies

#### Effect of pH

**Figure 7:**
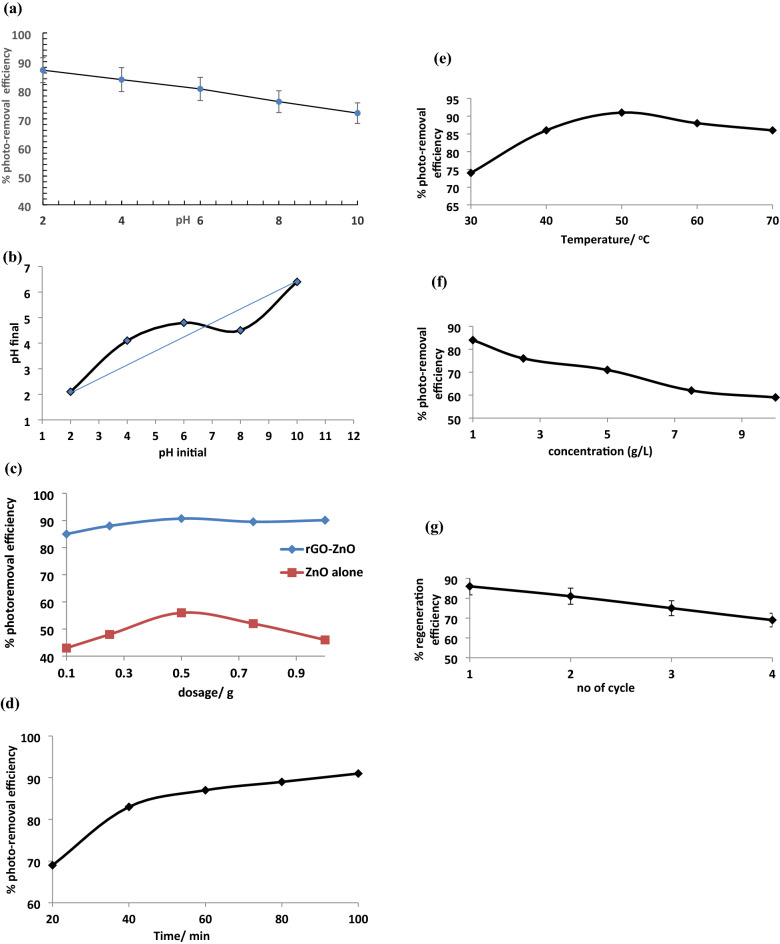
(**a**) Effect of pH on the photo-removal efficiency of CAP by rGO-ZnO nanocomposite, (**b**) pH_pzc_ of rGO-ZnO nanocomposite. (**c**) Effect of photocatalyst loading and composition on photo-removal efficiency of CAP. (**d**): Effect of irradiation time on the photo-removal efficiency of CAP. (**e**): Effect of temperature on the photo-removal efficiency of CAP. (**f**): Effect of CAP concentration on the photo-removal efficiency of CAP. (**g**): Regeneration efficiency of rGO-ZnO nanocomposite for the degradation of CAP.

The pH of the medium plays a vital role in the photodegradation process as it affects the surface charge of photocatalyst, semiconductor valence and conduction bands and CAP. The change in surface charge may influence the overall photocatalytic adsorption process by facilitating precipitation, co-precipitation and sorption processes occurring via electrostatic or hydrogen bonding interaction between the rGO-ZnO photocatalyst adsorbent and CAP molecules at the surface. From Fig. 7a, the % photo-removal efficiency decreased steadily from 88 to 72% as the pH increased from 2 (highly acidic) to 10 (alkaline). This is likely due to the fact that CAP molecule undergoes dissociation at higher pH values and non-dissociated form is more efficiently adsorbed onto rGO-ZnO nanocomposite surface. The pH_pzc_ (point of zero charge; the pH at which the surface charge is zero) of the nanocomposite photocatalyst was 4.80 (Fig. 7b). Below the pH_pzc_, the surface charge is positive while it is negative beyond the pH_pzc_. According to Quiang and Adams^32^, the acid dissociation constant (p*K*a) of CAP is 11.03 and CAP molecules existed largely in neutral form in this study. Consequently, there is a little electrostatic attraction between the photocatalyst and CAP molecules. The prominent adsorption interaction of CAP onto rGO-ZnO nanocomposite surface at lower pH might be due to the hydrogen-bonding interaction. At low pH conditions, a hydrogen bond could be formed between N–H, –OH, –NO_2_ groups in CAP and –OH, –COOH, C = O groups on the rGO component of the photocatalyst^33^. Ionisation of acidic functional groups occurs at higher pH and H_2_O competes with CAP molecules for charged functional groups. This competition results in the weakening of the hydrogen-bonding interaction between CAP molecules and the nanocomposite photocatalyst and hence the decline in photocatalytic adsorption capacity at higher pH. This result agreed with previous studies on the influence of pH on CAP removal using activated carbon^34,35^.

#### Effect of photocatalyst loading and composition

The effect of photocatalyst loading on the photo-removal efficiency of CAP was studied between 0.1 and 1.0 g at 40 °C, 1.0 g/L 40 mL CAP solution for 40 min in the presence of H_2_O_2_. From Fig. 7c, A steady increase in % photo-removal efficiency was observed as the rGO-ZnO nanocomposite dosage amount increased gradually from 0.1 g (85.1%) to its optimum value of 90.8% at 0.5 g. The increment might be attributed to the increase in the number of catalytic active sites on the rGO-ZnO nanocomposite surface resulting in the generation of greater amount of electron–hole pairs and hence the free radical species (*OH) necessary for the photocatalytic degradation of CAP molecules. At 0.5 g, equilibrium is attained and further increase in the photocatalyst concentration resulted in no net increase in the % photo-removal efficiency. Eskandari et al.^35^ reported an increase in photodegradation efficiency of ciprofloxacin from about 50% to 90% as zinc oxide nanoparticles photocatalyst increased from 0.05 to 1.5 g/L. The effect of photocatalyst composition was investigated using zinc oxide nanoparticles alone. Optimum % photo-removal efficiency of 56% at 0.5 g was achieved as against 90.8% in the case of rGO-ZnO nanocomposite under similar reaction conditions. It can be deduced that the incorporation of reduced graphene oxide into ZnO matrix leads to significant enhancement in photodegradation process of CAP by reducing band gap, preventing recombination of photogenerated electron–hole pairs and photocorrosion of the surface. This is in agreement with our previous work involving the photocatalytic degradation of Janus green dye by reduced graphene oxide Ag nanocomposite^36^.

#### Effect of irradiation time

Photocatalyst absorbs light with energy equal to or greater than the band gap energy. This results in the transfer of electrons from valence band to conduction band thereby leaving holes in the valence band. From Fig. 7d, the photocatalytic degradation of CAP increased with increasing UV irradiation time from 68% at 20 min and attained an optimum percentage photo-removal efficiency of 90% after 100 min. This might be due to the fact that the formation of photogenerated charge carriers (hole-electron) becomes facile at longer irradiation time leading to increase in the photo-removal efficiency of CAP. Abbasi^37^ reported enhancement in the photocatalytic removal efficiency of methyl orange upon increasing the irradiation time using magnetic (Fe_3_O_4_) graphene oxide (GO) and Fe_3_O_4_-GO-ZnO nanocomposites.

#### Effect of temperature

The temperature of the reaction medium played a crucial role in the photocatalytic degradation process. From Fig. 7e, a steady rise in % photo-removal efficiency of CAP was observed from 71% at 30 °C to 91% at 50 °C. Above 50 °C, a decline in photo-removal efficiency was recorded. The initial increase at temperatures below 50 °C might be attributed to increase in production of bubbles in the solution indicating the formation of free radicals thus accelerating the oxidation rate of CAP molecules at the interface. However, at higher temperature (above 50 °C), there is a lower saturation value of oxygen, a critical factor which regulates the photocatalytic mechanism by capturing the photogenerated electrons, as well as the increase of the desorption of the reactants from the catalyst surface, hence, the reduction in % photo-removal efficiency of CAP^38,39^.

#### Effect of CAP concentration

Photocatalytic degradation experiments were conducted between 1 and 10 g/L CAP concentration with rGO-ZnO photocatalyst dosage of 0.1 g at pH 2, 40 °C, for 40 min in the presence of H_2_O_2_ (Fig. 7f). Maximum % photo-removal efficiency (85.72%) was achieved at 1.0 g/L. Above 1.0 g/L, there is a gradual decline in photo-removal efficiency which might be due to the oversaturation of the active sites of the photocatalyst by CAP molecules thus preventing surface penetration by incoming UV radiation and hindering the generation of photons and electron–hole pairs. Consequently, there is limited population of much needed free radicals for degradation of CAP at higher concentrations. Similar observation was reported from previous studies involving ciprofloxacin using zinc oxide nanoparticles^40^.

#### Regeneration efficiency

Reusability and stability are key parameters often considered in the choice of suitable photocatalysts for many practical applications. The plot of the regeneration efficiency of rGO-ZnO photocatalyst on the degradation of CAP obtained for four consecutive cycles is presented in Fig. 7g. Percentage regeneration efficiency decreased from 87% after the first cycle to around 68% after the fourth cycle indicating the relative stability of the nanocomposite photocatalyst under prevailing conditions. This might be attributed to the reduced graphene oxide content of the nanocomposite which conferred greater adsorption capacity and faster charge separation for facile degradation of CAP pollutant.

### Adsorption isotherms and kinetic models of photocatalytic degradation of CAP

**Figure 8:**
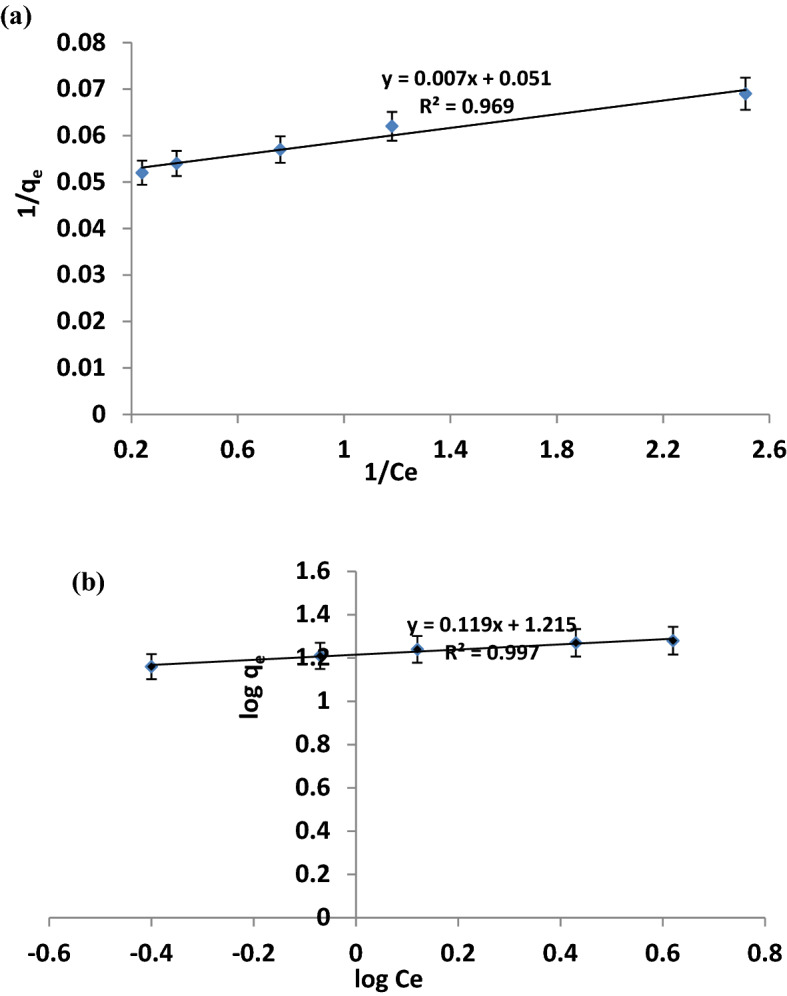
Adsorption isotherm plot for: (**a**) Langmuir model (**b**) Freundlich model.

Adsorption models provide detailed understanding of the nature of surface interaction occurring between the adsorbate (CAP) and the photocatalyst adsorbent. Both Langmuir and Freundlich adsorption isotherm models for the photodegradation of CAP by rGO-ZnO nanocomposite are presented in Fig. 8. The photocatalytic adsorption process fitted more accurately into the Freundlich adsorption model based on the regression values (r^2^ = 0.99) implying a multilayer adsorption mechanism of CAP onto the rGO-ZnO nanocomposite surface. The separation factor (*R*_*L*_*);* a useful non-dimensional parameter in isotherm studies was determined. *R*_*L*_ described the suitability of the nanocomposite and its affinity towards CAP removal. *R*_*L*_ value is obtained from Eq. (7):^25^

7 $$ R_{L}  = {{1}}/({{1}} + K_{L} C_{o} ) $$

where C_o_ is the initial concentration and *K*_*L*_ signifies the Langmuir constant. Four probabilities exist for the value of R_L_: *R*_*L*_ > 1.0, *R*_*L*_ = 1, 0 < *R*_*L*_ < 1, and *R*_*L*_ = 0, indicating unfavorable, linear, suitable, and irreversible degrees, respectively. From Table 1, the calculated R_L_ value of 0.0036 indicates the suitability of the adsorption process using rGO-ZnO nanocomposite.

**Table 1:** Adsorption isotherms, kinetic and separation factor parameters.

Adsorbate	Langmuir isotherm model constants	Freundlich isotherm model constants	Separation factor (R_L_)	Pseudo-1st order kinetic rate (/min^−1^)	Pseudo-2nd order kinetic rate (/mol^−1^L s^−1^)
CAP	K_L_ = 2800 q_m_ = 19.6 mg/g R^2^ = 0.96	K_F_ = *11.82 n = 8.40 R^2^ = 0.99	0.0036	k_1_ = 0.005 R^2^ = 0.97	k_2_ = 0.0034 R^2^ = 0.86

**Figure 9:**
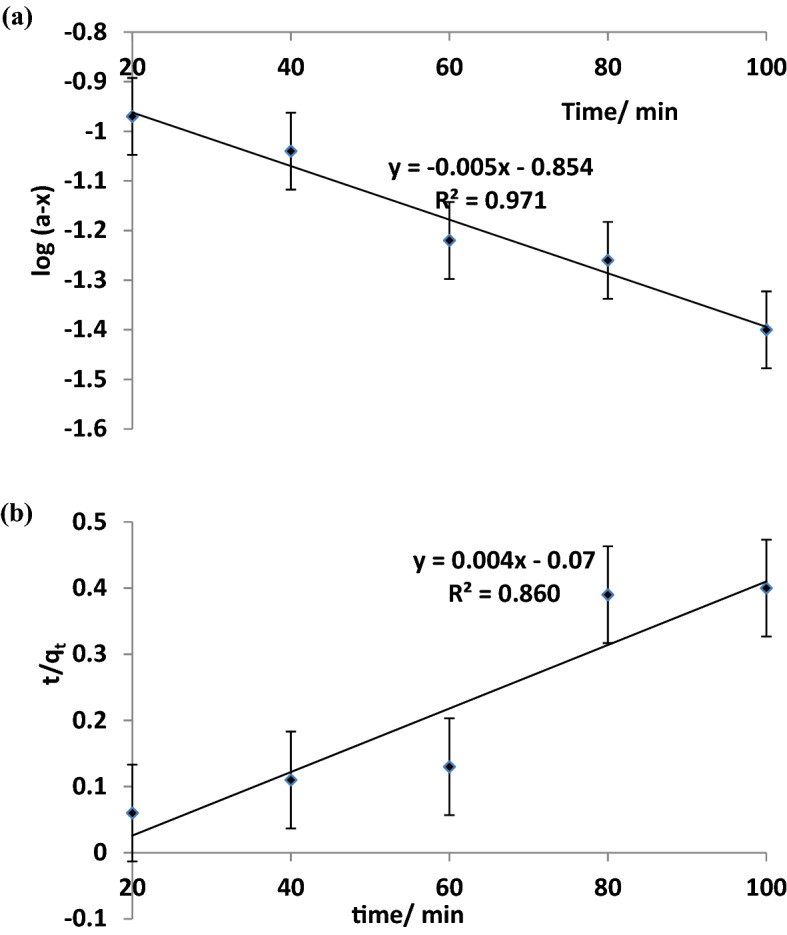
Pseudo-first (**a**) and second (**b**) order kinetic plots.

Figure 9 showed the result of the kinetic model studies. The rate of photocatalytic adsorption of CAP onto the nanocomposite surface is relatively faster with the pseudo-first order (0.005 min^−1^) than the pseudo-second order kinetics (0.0034 mol^−1^L min^−1^). It is noteworthy that with respect to the regression values of the kinetic studies (Table 1), the adsorption processes conformed more to the pseudo-first order kinetics implying a favourable physisorption mechanism for the photocatalytic degradation of CAP. Table 2 showed the comparative evaluation of CAP photocatalytic adsorption using rGO-ZnO nanocomposite and other adsorbents previously reported in literatures using Langmuir maximum adsorption capacity parameter. The rGO-ZnO nanocomposite of this study displayed a relatively higher adsorption capacity of CAP compared to most other adsorbents from previous studies. In particular, the adsorption capacity of rGO-ZnO nanocomposite (19.6 mg/g) was more than twice larger than that of bamboo charcoal (8.1 mg/g) and slightly greater than MIP based on magnetic chitosan microsphere (17.0 mg/g). However, it is much less than MWCNT adsorbent.

**Table 2:** Comparison of CAP adsorption capacity using different sorbent media.

Adsorbent material	Adsorption capacity (mg/g)	Reference
rGO-ZnO nanocomposite	19.6	This study
Bamboo activated charcoal	8.1	^41^
Molecularly imprinted polymer (MIP) based on magnetic chitosan microsphere	17.0	^42^
Multi-walled carbon nanotube (MWCNT)	107.9	^43^

### Treatment of veterinary effluent

The effectiveness of rGO-ZnO nanocomposite photocatalyst was investigated in the treatment of CAP laden veterinary effluent. Optimum % photo-removal efficiency of 90.2% for CAP and 92.74% reduction in the COD value (from initial value of 494.33 ± 0.69 mg/L to 35.91 ± 0.18 mg/L) were obtained upon the treatment of the effluent with rGO-ZnO nanocomposite thereby affirming its excellent photocatalytic degradation efficiency under prevailing conditions. The result of this work is comparable to previous studies on treatment of effluents using different photocatalyst adsorbents^3,36^.

### Proposed mechanism of rGO-ZnO photocatalytic degradation of CAP

**Figure 10:**
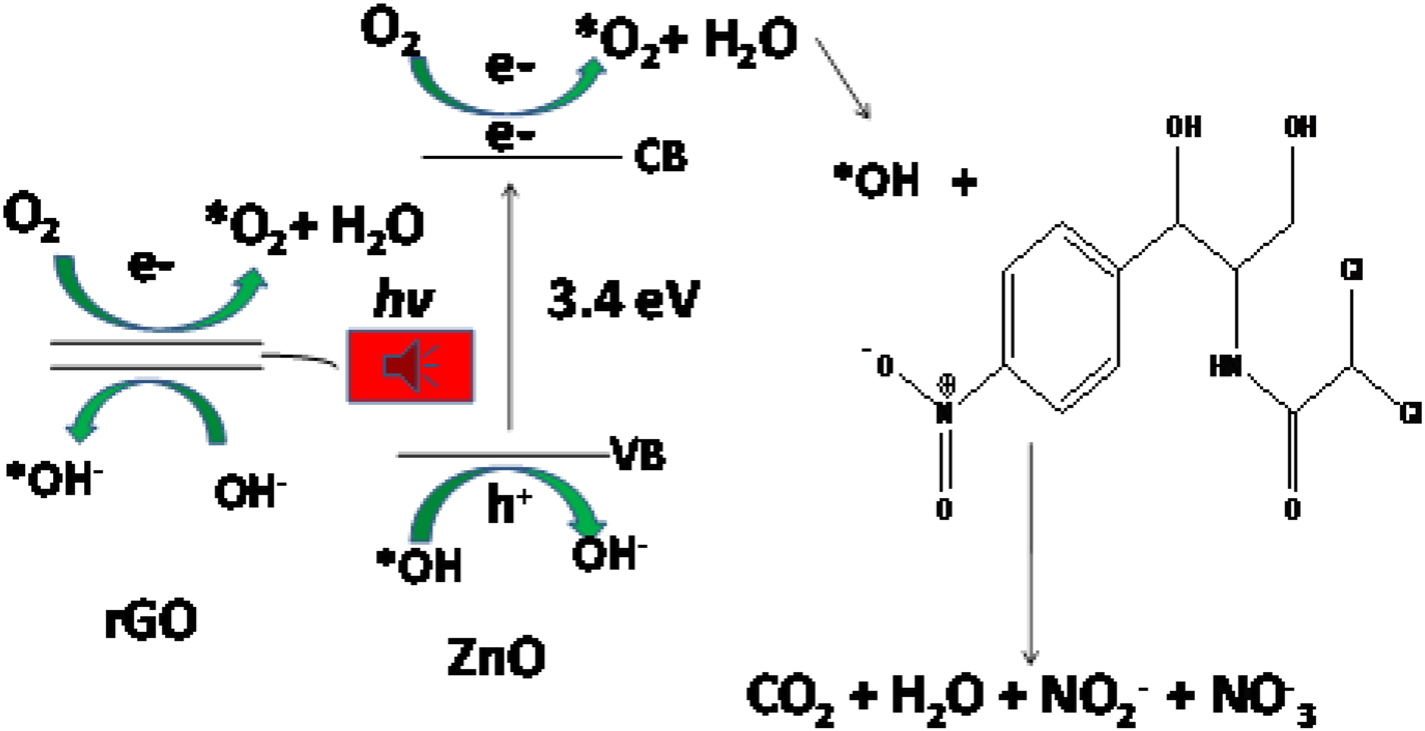
Proposed mechanism of photocatalytic degradation of CAP by rGO-ZnO nanocomposite.

Interaction of the rGO-ZnO nanocomposite photocatalyst with UV radiation and H_2_O_2_ result in the excitation of electron from the valence band to the conduction band of the ZnO semiconductor of the nanocomposite and the generation of electron–hole pairs (Fig. 10). The reduced graphene oxide facilitates the reduction of the initial band gap and thus promotes rapid migration of charges and generation of free hydroxyl radicals (*OH) upon interaction of OH^-^ ions (from water) with the positive holes (h +) of the valence band of ZnO. The conductive band electron reacts with O_2_ forming a superoxide radical which further reacts with water to yield hydroxyl radicals. The generated hydroxyl radicals oxidize chloramphenicol molecule to water, CO_2_, with nitrate and nitrite as by-products.

## Conclusion

A green and economical synthesis of rGO-ZnO nanocomposite using waste battery graphite rod powder and *A. cruentus* aqueous extract as GO precursor and reducing agent respectively was successfully developed. Phytochemical screening of the *A. cruentus* aqueous extract confirmed the presence of terpenoids, flavonoids, saponins and carbohydrates which were responsible for the capping, bioreduction and stabilization of the nanocomposite. TEM morphology revealed a polydispersed, quasi-spherical zinc oxide on a rough, coarse reduced graphene surface. XRD patterns of the synthesized rGO-ZnO nanocomposite showed sharp, prominent crystalline wurtzite hexagonal phases of ZnO and rGO. Presence of hydrogen peroxide, lower temperatures and pH as well as prolonged exposure to UV irradiation favoured adsorptional photocatalytic degradation of CAP. Higher optimal % photo-removal efficiency of 91% was achieved with rGO-ZnO nanocomposite compared to 56% for ZnO nanoparticles alone under similar conditions. The presence of reduced graphene oxide in the composite facilitated easy generation and separation of electron–hole pairs, improved adsorption capacity and conductivity. Photocatalytic adsorption process followed more accurately the pseudo-first order rate law and Freundlich model implying a multilayer mechanism. The rGO-ZnO nanocomposite displayed a relatively better photocatalytic activity and stability than most of the previously reported adsorbents and was successfully applied in the treatment of CAP laden veterinary effluent.
